# Skewing in Arabidopsis roots involves disparate environmental signaling pathways

**DOI:** 10.1186/s12870-017-0975-9

**Published:** 2017-02-01

**Authors:** Eric R. Schultz, Agata K. Zupanska, Natasha J. Sng, Anna-Lisa Paul, Robert J. Ferl

**Affiliations:** 1 Department of Horticultural Sciences, Program in Plant Molecular and Cellular Biology, University of Florida, Gainesville, FL 32611 USA; 2 Interdisciplinary Center for Biotechnology Research, University of Florida, Gainesville, FL 32610 USA; 3 Present address: Department of Biology, Washington University in St. Louis, St. Louis, MO 63130 USA

**Keywords:** Transcriptomics, Root skewing, Root waving, Morphometrics, Microarray, *Arabidopsis thaliana*

## Abstract

**Background:**

Skewing root patterns provide key insights into root growth strategies and mechanisms that produce root architectures. Roots exhibit skewing and waving when grown on a tilted, impenetrable surface. The genetics guiding these morphologies have been examined, revealing that some Arabidopsis ecotypes skew and wave (e.g. WS), while others skew insignificantly but still wave (e.g. Col-0). The underlying molecular mechanisms of skewing and waving remain unclear. In this study, transcriptome data were derived from two Arabidopsis ecotypes, WS and Col-0, under three tilted growth conditions in order to identify candidate genes involved in skewing.

**Results:**

This work identifies a number of genes that are likely involved in skewing, using growth conditions that differentially affect skewing and waving. Comparing the gene expression profiles of WS and Col-0 in different tilted growth conditions identified 11 candidate genes as potentially involved in the control of skewing. These 11 genes are involved in several different cellular processes, including sugar transport, salt signaling, cell wall organization, and hormone signaling.

**Conclusions:**

This study identified 11 genes whose change in expression level is associated with root skewing behavior. These genes are involved in signaling and perception, rather than the physical restructuring of root. Future work is needed to elucidate the potential role of these candidate genes during root skewing.

**Electronic supplementary material:**

The online version of this article (doi:10.1186/s12870-017-0975-9) contains supplementary material, which is available to authorized users.

## Background

Plant roots have been long studied and yet investigations of root behavior, physiology, and biochemistry continue to be actively explored on every level [1–6]. The work presented here seeks further insights into root growth strategies by focusing on two distinct growth patterns of root growth, skewing and waving. Skewing is when the steady-state growth direction of a root deviates from the direction of the gravity vector and waving is when the root undulates back and forth over time along its direction of growth [7–14]. Skewing and waving have been described as gravity-enhanced touch responses, since these root patterns are notably present when a plant is grown on a tilted, impenetrable surface. Current models require that gravity pulls the root tip down onto the tilted surface, which increases the mechanical impedance to growth, and results in root skewing and root waving [15]. However, recent spaceflight experiments with *Arabidopsis thaliana* (Arabidopsis) on the International Space Station (ISS) have shown that certain ecotypes have roots that deviate from vertical growth (at a magnitude similar to skewing on the ground) and wave in the absence of gravity [16–18]. These ecotypes that exhibit spaceflight skewing are also the ecotypes that exhibit terrestrial skewing when grown at an angle [16–18]. The degree of skewing in spaceflight was consistent with previous terrestrial observations, but the waving pattern was different from what was typically observed on the ground [17]. The causes of skewing – and subsequently, the genes involved – are currently unknown. The goal of this study is to discover which genes are associated with root skewing through transcription microarrays.

Other root tropisms exist as a result of environmental interaction, which likely impact the degree of root skewing and root waving. Touch responses, or thigmotropism, involves many genes that interact with auxin and can subsequently alter growth patterns [11, 19–28]. Plants also determine their growth in relation to the gravity vector, resulting in a gravitropic set-point angle (GSA), which is most commonly associated with lateral organ growth relative to the primary organ [29]. GSA is driven by auxin and TRANSPORT INHIBITOR RESPONSE 1/AUXIN SIGNALING F-BOX (TIR1/AFB)-dependent signaling, in a process similar to gravitropism [30–32]. Light can alter GSA for different plant systems [33], and light in general plays a role in root patterning and growth direction [34–42]. Additional tropisms include halotropism [39, 43, 44], chemotropism [45], hydrotropism [46], all of which can alter root growth response (reviewed in [3]).

Many hypotheses exist for the underlying molecular mechanisms and key genes responsible for root skewing and root waving. As a result, a wide variety of genes have been implicated as involved in these growth patterns. Hormone related pathways and processes are among the candidates, such as ethylene involvement in root waving [10] and auxin and tryptophan biosynthesis in both skewing and waving [47, 48]. The cytoskeleton is also involved in skewing and waving [7, 18, 49, 50], including *WAVE-DAMPENED 2* (*WVD2*; At5g28646) and *WVD2-LIKE 1* (*WDL1*; At3g04630) genes, which alter cell expansion through microtubule bundle organization [51]. Many genes involved in molecular patterning, signaling, phosphorylation, and cell wall structure are also involved in root skewing and waving. Guanosine triphosphate (GTP)-binding proteins [11], cell expansion gene *ROOT HAIR DEFECTIVE 3* (*RHD3*; At3g13870) with putative GTP-binding motif [13], *CLAVATA*-related genes [52], protein phosphatase/PP2A *ENHANCED ETHYLENE RESPONSE 1* (*RCN1*; At1g25490; [53]), serine/threonine protein kinases *WAG1* and *WAG2* (At1g53700 and At3g14370, respectively; [54]), and *KNOTTED-LIKE FROM ARABIDOPSIS THALIANA 1* (*KNAT1*; At4g08150; [55]) are all associated with morphological change in root skewing or root waving. The SKU family of genes were identified through phenotypic changes in root behavior, with *SKU5* (At4g12420) and related genes being involved with cell wall modification and root growth [56] and *SKU6* (At2g03680) and family being involved with cortical microtubule and directional cell expansion [50]. While no single pathway has been identified in the literature as responsible for root skewing or for root waving, several different pathways have evidence for being involved.

Skewing and waving are functionally separable using genetics [7, 12, 13, 51, 57]. The physiological separation of skewing from waving within a ecotype can be accomplished by using the environment, particularly the tilt angle of the growth plate, and can also occur in spaceflight [16–18]. The degree to which growth plates are tilted is referred to as the Angle of the growth plate (A_gp_). Typically, plates are tilted backward such that plants are above the media surface, at A_gp_ 45°. For many Arabidopsis ecotypes, growth at A_gp_ 45° results in roots that both skew and wave. When growth plates are tilted forward, such that plants are below the media surface, at A_gp_ 135°, Arabidopsis roots skew with reduced waving, similar to the pattern observed in spaceflight. Growth plates held vertically are A_gp_ 90° and are considered as the controls for growth angle, as roots on vertical plates generally do not skew or wave.

This study aims to discover additional genes associated with root skewing through transcription microarray analyses of Arabidopsis roots exhibiting different patterns of waving and skewing. Gene expression profiles were derived from Arabidopsis that displayed different root growth behaviors in various growth environments in order to identify gene activities associated with skewing and waving. Identified genes were validated using qRT-PCR and evaluated using previous studies for likelihood of involvement with the skewing process.

## Results

### Arabidopsis ecotypes skew and wave differently from one another in different growth environments

**Fig. 1:**
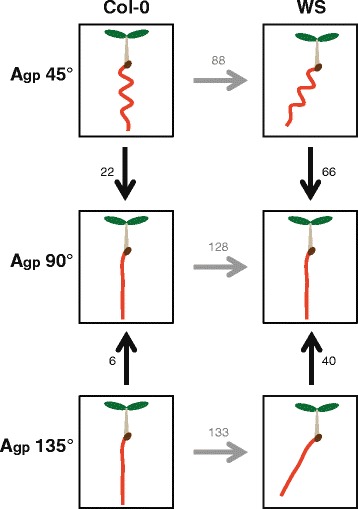
Various comparisons of microarray data reveals different genes involved in root skewing and waving. *Arrows point* to control used in each experiment. *Vertical arrows* indicate comparisons within ecotypes, horizontal arrows indicate comparisons between ecotypes. Eight-day experiment designed to isolate each permutation of root skewing and root waving and identify genes responsible. Vertical comparisons reveal genes responsible for changing the root growth pattern in response to different A_gp_ – for WS, these genes correlate to skewing and waving phenotypes. Horizontal comparisons reveal genes responsible for differences in skewing and waving for Col-0 and WS roots. Numbers indicate gene transcripts with different levels of transcripts from controls. Significance cutoffs of |log_2_(FC)| > 1, *p* < 0.05

**Fig. 2:**
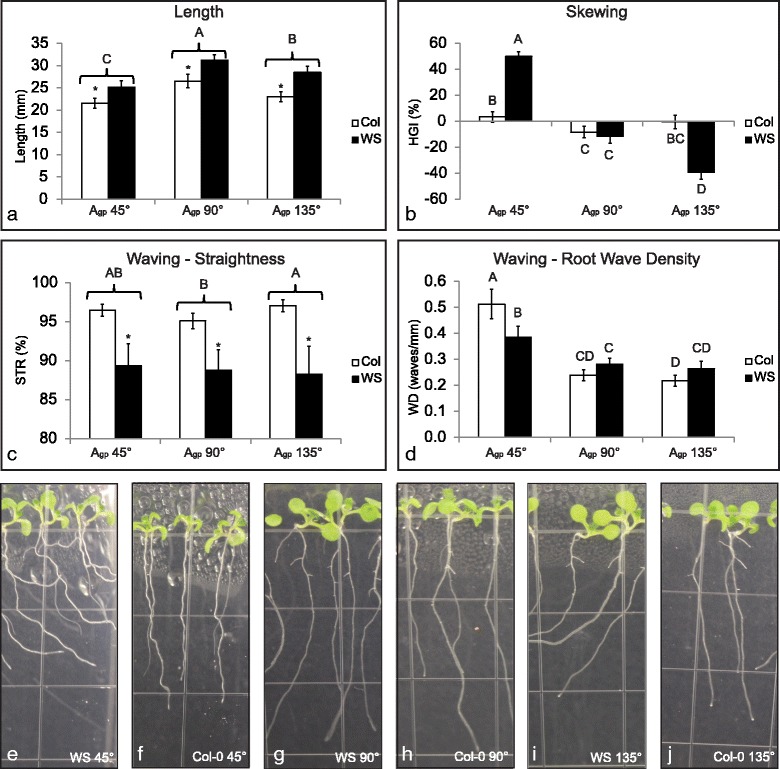
WS roots grown at A_gp_ 135° skew with reduced waving, while WS roots grown at A_gp_ 45° both skew and wave. Plants were grown vertically for 3 days on 0.5% Phytagel agar media, moved to their respective growth conditions, and grown for 5 more days. Plants were imaged, harvested, and fixed in RNAlater 8 days after germination (5 days after gravistimulation). Bars represent means (average *n* = 43) and error bars represent 95% confidence interval. Measurements were conducted using ImageJ plugin JFilament [106–108] and were processed using custom R scripts available on GitHub [109]. For comparisons where interaction of the two independent variables is significant, results of Scheffé’s method are represented with letters. Bars with different letters are different from one another (*p* < 0.05). For comparisons where interaction of the two independent variables is not significant, brackets and stars are used in addition to letter codes (*p* < 0.05 = *). Brackets indicate grouped measurements; all bars for one unit on the x-axis are compared to other bars for other x-axis units, and are represented by the significance indicator above the bracket. **a** Length of primary root. Roots grown at A_gp_ 90° were the longest, followed by roots grown at A_gp_ 135°, and roots grown at A_gp_ 45° were the shortest. Col-0 roots were shorter than WS roots. **b** Horizontal growth index of primary root. WS roots skewed more than Col-0 roots when grown at A_gp_ 45° and 135°. **c** Straightness of primary root. WS roots were less straight than Col-0 roots. **d** Root wave density. Roots grown at A_gp_ 45° had the highest WD. **e** Representative image of WS at A_gp_ 45°. **f** Representative image of Col-0 at Agp 45°. **g** Representative image of WS at A_gp_ 90°. **h** Representative image of Col-0 at A_gp_ 90°. **i** Representative image of WS at A_gp_ 135°. **j** Representative image of Col-0 at A_gp_ 135°. All images (courtesy of author) were taken through media

Arabidopsis ecotypes Wassilewskija (WS) and Columbia (Col-0) differ in their skewing and waving behaviors, where WS demonstrates strong skewing and waving while Col-0 waves like WS but does not greatly skew, which was a growth pattern observed in spaceflight and on the ground (selected references: [14, 17, 49]). These two ecotypes were grown at three growth angles (A_gp_ 45°, 135°, or vertical control of A_gp_ 90°) to produce different root phenotypes (Figs. 1 and 2). Plants were grown for 3 days vertically, then moved to each respective A_gp_ for 5 more days, resulting in steady-state behavior in each of the A_gp_.

The first analysis compared transcriptional differences within a ecotype caused by the environmental condition of A_gp_, specifically differences between WS roots grown at A_gp_ 90° and WS roots grown at A_gp_ 45° or 135°, and the differences between Col-0 roots grown at A_gp_ 90° and Col-0 roots grown at A_gp_ 45° or 135°. Only the A_gp_ influenced the differences in gene expression and root morphology. Roots were quantified following parameters identified in previous work [12, 58] using protocols outlined in the Materials and Methods section. The arrows in Fig. 1 represent comparisons analyzed. Figure 1 also shows a diagram of the root morphology for each genotype under each A_gp_.

The second analysis compared transcriptional differences between ecotypes at each A_gp_ (e.g. differences between Col-0 and WS roots grown at A_gp_ 45°). In this case, the ecotype influenced gene expression and morphology at the given A_gp_. The horizontal arrows in Fig. 1 show these comparisons.

This matrix of comparisons allows two overlapping approaches to using differential expression to identify genes associated with skewing. WS roots skew significantly more than Col-0 roots at A_gp_ 135° and significantly more than WS roots at A_gp_ 90° (see Fig. 1). At A_gp_ 45° WS roots skew more than Col-0 roots, but both WS and Col-0 roots also wave at 45°. Quantification of root morphometrics is found in Fig. 2, using horizontal growth index (HGI; trigonometric relationship between the overall angle of growth and length of the root), straightness (STR; straight-line length of the root from start point to end point divided by the actual length of the root), and wave density (WD; waves per millimeter along root length). Comparisons involving A_gp_ 45° allow the removal of waving as a confounding factor in the gene expression analyses.

### Col-0 roots showed 24 significantly altered transcripts related to A_gp_, most of which may be involved in waving.

These comparisons are represented by the vertical arrows in the left column of Fig. 1, with the numbers indicating the number of significantly altered transcripts from each comparison (significance cutoffs of |log_2_(fc)| > 1; *p* < 0.05). False discovery rate (FDR) corrections are found in Table 1.

**Table 1:**
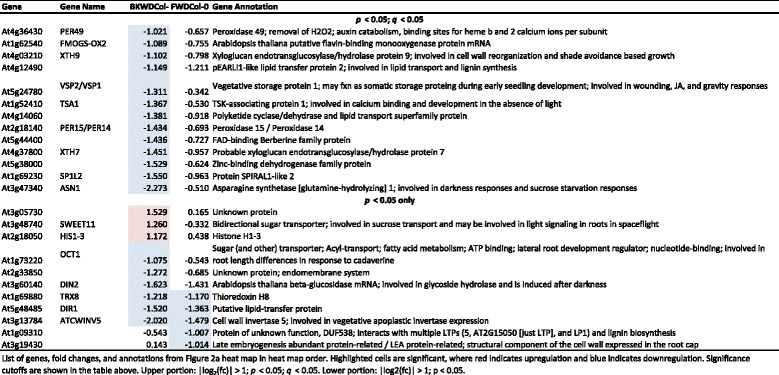
Comparing different growth angles to vertical within Col-0

**Fig. 3:**
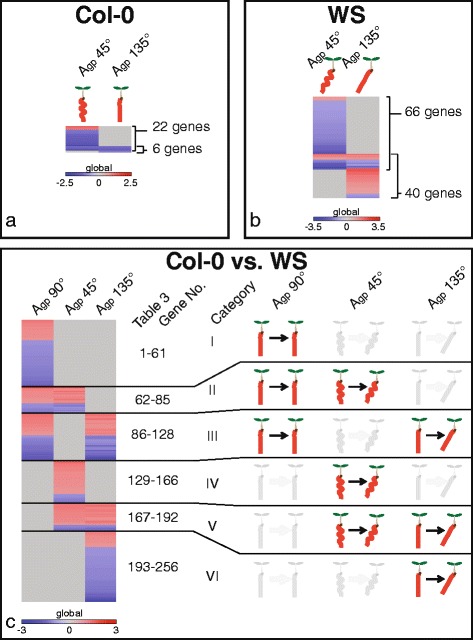
Comparison of transcriptomes as they relate to morphology. Heat maps illustrate differences generated by angle of the growth plate (A_gp_) or genetic differences. **a** Genes with altered transcription within Col-0 compared to the control of A_gp_ 90°. Col-0 had 22 gene transcripts altered at A_gp_ 45 ° and 6 altered at A_gp_ 135°. Data in the heat map corresponds with data in Table 1. **b** Genes with altered transcription within WS compared to the control of A_gp_ 90°. WS had 66 altered gene transcripts at A_gp_ 45° and 40 altered at A_gp_ 135°. Data in heat map corresponds with data in Additional file 1: Table S2. **c** Transcript comparisons between Col-0 and WS ecotypes – a genetic comparison – with morphological diagrams corresponding to each of the categories (I-VI) of gene expression profile. Transcripts between the ecotypes were removed if they were altered at the same level at all A_gp_. For example, if the transcript for *Gene A* was downregulated 2 fold in all conditions, it was removed. This comparison between Col-0 and WS had 128 altered gene transcripts at A_gp_ 90°, 88 altered between Col-0 and WS at A_gp_ 45°, and 133 altered between Col-0 and WS at A_gp_ 135°. Data in heat map corresponds to data in Additional file 2: Table S3.

When gene expression profiles of roots grown at A_gp_ 45° were compared to A_gp_ 90°, 22 genes were shown to be differentially regulated, three of which were upregulated and 19 of which were downregulated (Fig. 3a). Col-0 plants grown at A_gp_ 45° had roots that waved but did not skew as compared to the roots of plants grown at A_gp_ 90°. Thus these 22 differentially expressed genes were associated with a root waving and but not root skewing.

Comparison of gene expression profiles of roots grown at A_gp_ 135° with profiles of roots grown at A_gp_ 90° (Fig. 3a), revealed only 6 genes that were differentially regulated between the two growth conditions. All 6 of these genes were downregulated. Morphologically, Col-0 roots grown at A_gp_ 135° were not significantly different from those grown at A_gp_ 90° (Fig. 2). Four genes out of these six were also present among the significantly differentially expressed genes in the A_gp_ 45° comparison to A_gp_ 90°, with just 2 being unique (At1g09310; a protein of unknown function with DUF538 and At3g19430; a protein related to late-embryogenesis abundant proteins, Table 1).

Thus the 18 genes unique to the A_gp_ 45° comparison to their 90° controls may represent genes associated with root waving, but not skewing. Many different processes were associated with these 18 genes identified, such as cell wall structure (*XTH7*, *XTH9*) and sugar transport (*SWEET11*, *OCT1*; Table 1).

Only four genes had altered transcript levels at both A_gp_ 45° and 135°, meaning that they responded to both the backward and forward tilted growth environments (Fig. 3a). All four of these genes were downregulated at approximately the same levels in both conditions. These genes were *At4g12490* (a pEARLI1-like LTP), *THIOREDOXIN H8* (*TRX8*; At1g69880), *DEFECTIVE IN INDUCED RESISTANCE 1* (*DIR1*; At5g48485; putative LTP), and *CELL WALL INVERTASE 5* (*ATCWINV5*; At3g13784).

### WS showed 92 significantly altered transcripts related to A_gp_, revealing candidates genes involved in skewing

These comparisons are represented by the vertical arrows in the right column of Fig. 1. The number of genes with significantly altered transcripts is located next to the appropriate arrow in Fig. 1 (significance cutoffs of |log_2_(fc)| > 1; *p* < 0.05). False discovery rate (FDR) corrections are found in Additional file 1: Table S2.

At A_gp_ 45°, WS produces roots that both skew and wave compared to the roots grown vertically (A_gp_ 90°). Gene expression profiles revealed that WS that were grown at A_gp_ 45° had 66 genes with altered transcription in roots compared to A_gp_ 90°. Nine of the 66 genes were upregulated, while 57 were downregulated (Fig. 3b and Additional file 1: Table S2). These genes may represent those involved in both skewing and waving processes. Processes associated with these genes include lipid transfer (*AZELAIC ACID INDUCED 1* [*AZI1*; At4g12470], *CELL WALL-PLASMA MEMBRANE LINKER PROTEIN* [*CWLP*; At3g22120]), pectin methylesterase inhibition (At5g62330, At2g01610), and peroxidase activity (*PEROXIDASE 49* [*PER49*; At4g36430], *PEROXIDASE 53* [*PER53*; At5g06720]; Additional file 1: Table S2).

When grown at A_gp_ 135° WS produces roots that skew without waving (Fig. 2). Gene expression profiles revealed that WS roots grown at Agp 135° had 40 genes with altered transcription in roots compared to WS roots grown at A_gp_ 90°. These 40 genes are likely skewing-related (Fig. 3b). Only two genes (*HISTONE H1-3* [*HIS1-3*; At2g18050] and *DARK INDUCIBLE 2* [*DIN2*; At3g60140]) also had differential transcription in Col-0, and the expression level of *DIN2* was in opposite directions (Table 1 and Additional file 1: Table S2). Some of the processes included are methionine metabolism related (*METHIONINE GAMMA-LYASE* [*MGL*; At1g64660]), histone-related (*HIS1-3*), and jacalin-related (*JACALIN-RELATED LECTIN 40* [*JAL40*; At5g28520]). Other genes from these 40 will be discussed in more detail in the following sections.

The genes with altered transcription in both A_gp_ 45° and at A_gp_ 135° (14 out of the 40 genes previously mentioned) were altered in the same way in both A_gp_ 45° and 135° compared to A_gp_ 90°, except for one gene. This one gene was *ASPARAGINE SYNTHETASE [GLUTAMINE-HYDROLYZING] 1* (*ASN1*; At3g47340, also called *DIN6*,), which was downregulated in A_gp_ 45° and upregulated in A_gp_ 135°, and is involved in darkness responses and sucrose starvation responses. The remaining 13 genes were involved in various processes, including cell wall reorganization (*XYLOGLUCAN ENDOTRANSGLUCOSYLASE/HYDROLASE 9* [*XTH9*; At4g03210]) and sugar transport (*SWEET11* and *SWEET12*; At3g48740 and At5g23660, respectively).

### Transcriptome comparisons between ecotypes revealed genes that could be involved in skewing in WS

**Fig. 4:**
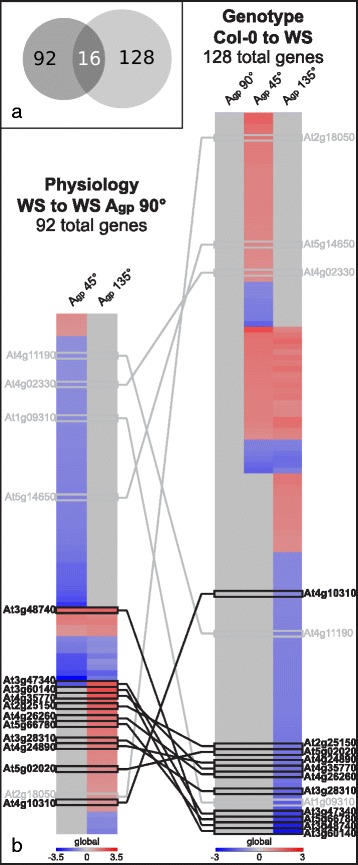
Comparison of candidate skew genes from physiological and genotypic comparisons. **a** Venn diagram of gene numbers from Fig. 3, illustrating overlap of 16 genes. The left circle represents total number of genes changed in WS (Fig. 3b) and the right circle represents total number of genes changed between Col-0 and WS in groups IV, V, and VI (Fig. 3c). **b** Genes with altered transcription identified in Fig. 3b (*left column*) were combined with genes with altered transcription identified in groups IV, V, and VI of Fig. 3c (*right column*), resulting in 16 genes. The majority of overlapping gene transcripts occurs with A_gp_ 135° – a growth condition that elicited root skewing with minimal waving – totaling 11 highly probable skew gene candidates (HPSGC), shown here in bolded text with dark connecting lines

The gene expression profiles between Col-0 and WS when skewing and waving were also compared. These comparisons are represented in Fig. 1 by the horizontal arrows pointing toward WS, with the number of significantly altered levels of gene transcripts indicated above each arrow. Genes altered between Col-0 and WS at the same level in all three growth angles were removed from the comparison, as they represent inherent differences between the cultivars that were independent of the testing environments. The plant images and morphometrics are found in Fig. 2, with resulting heat map with six different patterns of expression pattern categories in Fig. 4 and gene annotations, information, and significance cutoffs in Additional file 2: Table S3.

The first differential gene expression pattern had altered transcripts between Col-0 and WS only when grown A_gp_ 90°. Genes 1–61 had altered transcript levels in Category I (Fig. 3c); 19 of the identified genes were upregulated and 42 of which were downregulated (Additional file 2: Table S3). This pattern indicated inherent genetic differences between these two lines in their control condition and these differences were mitigated or no longer present in other tested growth environments.

The second differential gene expression pattern had altered transcripts between Col-0 and WS when cultivars grown A_gp_ 90°, but also had altered transcripts in a same way when grown at A_gp_ 45°. Genes 62–85 had altered transcript levels in Category II (Fig. 3c); 15 of the identified genes were upregulated and 9 downregulated when comparing the gene expression in Col-0 to WS roots (Additional file 2: Table S3). This expression pattern was not considered to be involved with skewing, since A_gp_ 45° results in a combination of skewing and waving in WS and waving in Col-0. Additionally, the genes identified in this expression pattern were not significantly different in A_gp_ 135°, which generates skewing without waving in WS and a non-skewing, non-waving phenotype in Col-0 (Fig. 2).

The third expression pattern had altered transcript levels between Col-0 and WS when grown A_gp_ 90° and at A_gp_ 135°. Genes 86–128 had altered transcript levels in Category III (Fig. 3c); 20 of the identified genes were upregulated and 23 genes downregulated when comparing Col-0 to WS (Fig. 3c and Additional file 2: Table S3). At A_gp_ 135° WS skewed and Col-0 did not, yet these genes showed same differential expression between Col-0 and WS roots when grown at A_gp_ 90° and do not exhibit any skewing or waving differences (Figs. 2 and 3c). These genes were likely not involved with skewing.

The fourth expression pattern had altered transcript levels between Col-0 and WS only when both were grown at A_gp_ 45°. Genes 129–166 had altered transcript levels in Category IV (Fig. 3c); 30 of the identified genes were upregulated and 8 were downregulated (Fig. 3c and Additional file 2: Table S3). The genes identified represent a combination of skewing and waving phenomena, and as such, were considered as potential root skewing candidates.

The fifth expression pattern had altered transcript levels at both A_gp_ 45° and 135° when comparing Col-0 roots to WS roots. Genes 167–192 had altered transcript levels in Category V (Fig. 3c); 20 of the identified genes were upregulated and 6 were downregulated (Fig. 3c). Skewing occurred in WS but not in Col-0 in both conditions (Fig. 2), although at A_gp_ 45°, skewing occurred with waving. For WS roots, skewing was a distinguished morphological phenotype from Col-0. The genes identified had altered transcript levels in both A_gp_ 45° and 135°, and are therefore likely involved with skewing.

The sixth and final expression pattern had altered transcript levels at A_gp_ 135° only, when comparing Col-0 to WS. Genes 193–256 had altered transcript levels in Category VI (Fig. 3c); 14 of the identified genes were upregulated and 50 genes were downregulated, meaning that WS had higher levels of these 50 genes when compared to Col-0. These identified genes were likely involved in skewing since skewing occurs in WS at A_gp_ 135° with minimal waving but it does not in Col-0, thus is the only phenotypic difference between the roots of the two ecotypes (Fig. 2). The genes from the fifth and the sixth expression patterns, totaling 90 genes, were selected as a root skewing candidate genes.

### Overlap of skew gene candidates from all comparisons further narrows the set of skew gene candidates

When combining the lists of genes identified in Categories IV, V, and VI (128 genes) with genes identified within WS comparisons (66 genes altered at A_gp_ 45° and 40 genes altered at A_gp_ 135°), 16 genes are shared (Fig. 4). This overlap is illustrated by the Venn diagram in Fig. 4a. The majority of shared genes between the two data sets involved A_gp_ 135° – the growth condition that elicited skewing with minimal waving – resulting in 11 genes, which were named the highly probable skew gene candidates (HPSGC, Table 2; also indicated by “Y*” in Additional file 1: Table S2). Several of the HPSGC have been verified with qRT-PCR, showing similar trends to the transcriptomic microarray data [see Additional file 3]. The HPSGC are involved in a variety of cellular processes. Additionally, only 3 of these 11 genes were shared with the set of genes identified in Col-0 grown at A_gp_ 45°, which induced root waving (Fig. 3a, Table 1). Two of these genes (*DIN2* and *ASN1*) were expressed in opposite directions, while the remaining gene (*SWEET11*) was upregulated in all sets.

**Table 2:** HPSGC and their various functional groups and signaling pathways that could contribute to WS root skewing

Gene	Symbol	Log2(FC)	Catalytic activity	Hormone	Cell Wall	Sugar	Biotic stress	Mobile mRNA	Dark	Salt	Root cell	Water	SUBA3
*p* < 0.05; *q* < 0.05													
At4g26260	*MIOX4*	1.570	X	X		X					X		cytosol
At4g24890	*PAP24*	1.414	X		X								extracellular
At3g47340	*ASN1*	2.080	X			X			X				cytosol
At2g25150		1.839	X										cytosol
At5g02020	*SIS*	1.200								X			nucleus
At5g66780		1.556											plastid
At3g28310		1.430											nucleus
*p* < 0.05 only													
At3g60140	*DIN2*	2.214	X			X		X	X				vacuole, extracellular
At4g35770	*STR15/SEN1*	2.100		X					X				plastid (chloro., thylakoid)
At3g48740	*SWEET11*	1.804				X	X						plasma membrane
At4g10310	*HKT1*	1.021								X		X	plasma membrane

HPSGC are reported, with “X” indicating to which process or category each member belongs. SUBA3 reports localization of each HPSGC

The 11 genes comprising the HPSGC, indicated by bolded text in Fig. 4b, are *MYO-INOSITOL OXYGENASE 4* (*MIOX4*; At4g26260; involved in inositol oxygenase activity, syncytium formation, and iron ion binding), *PURPLE ACID PHOSPHATASE 24* (*PAP24*; At4g24890; involved in protein serine/threonine phosphatase activity), *SWEET11* (a sucrose efflux transporter), *DIN2* (a beta-glucosidase mRNA, involved in glycoside hydrolase, and is induced after darkness), *ASN1* (involved in darkness and sucrose starvation responses), *SENESCENCE 1* (*SEN1*; At4g35770; senescence-associated, induced by phosphate starvation), *HIGH AFFINITY K+ TRANSPORTER 1* (*HKT1*; At4g10310; sodium transporter in xylem parenchyma), *SALT INDUCED SERINE RICH* (*SIS*; At5g02020; involved in salt tolerance), At2g25150 (HXXXD transferase family protein involved in transferring acyl groups other than amino-acyl groups), At5g66780 (unknown gene), and At3g28310 (unknown gene containing DUF677).

## Discussion

### Transcriptome comparisons within ecotypes reveal a primary set of 92 candidate genes involved in skewing

The primary set of 92 candidate genes involved in skewing was identified by comparing the transcription of WS genes when grown at A_gp_ 45° or 135° compared to A_gp_ 90°. Morphologically, WS roots skew when grown at A_gp_ 135° with reduced waving (Figs. 1 and 2). The genes with altered transcription identified in this comparison of A_gp_ 135° to A_gp_ 90° represent the pool of genes likely involved in root skewing and not waving, since they were altered in a condition that induces root skewing independent of the classical root waving patterns. WS roots also skew when grown at A_gp_ 45°; however, the occurrence of waving at A_gp_ 45° complicates the relationship between skewing and transcriptome at A_gp_ 45°. It is important to note that only roots were used for the microarrays, and that lateral roots appeared to be similar across all treatments. As seen in Table 2, the 11 genes associated with skewing cluster into a few biological categories, suggesting that several pathways interact to produce skewed growth and directionality.

Col-0 roots, on the other hand, did not skew as did WS roots, and as such, differed only in waving when A_gp_ 45° or 135° were compared to A_gp_ 90°. The number of genes with altered transcription at A_gp_ 45° or 135° compared to A_gp_ 90° was also reduced, and not considered in the list of candidate skew genes due to the lack of skewing morphology.

### Transcriptome comparisons between ecotypes revealed a different set of 128 genes that may be involved in skewing

Comparing gene expression patterns between WS and Col-0 shows how the ecotypes cope with the same environmental challenges presented to them, in this case whether the angle of the growth plate produces skewed roots. In order to determine which gene expression patterns are related to skewing, the relative expression patterns can be correlated to the morphology generated in each of the growth angles.

Categories I-III in Fig. 3c are not related to skewing. The first category (Category I, Genes 1–61; Fig. 3c and Additional file 2: Table S3) contains genes that were different between WS and Col-0 at A_gp_ 90° and also unchanged at A_gp_ 45° or 135° (Additional file 2: Table S3). The second category (Category II, Genes 62–85; Fig. 3c and Additional file 2: Table S3) contained genes that were differentially expressed between WS and Col-0 at A_gp_ 45°. However, the transcript expression of these genes was also different when the roots did not skew at A_gp_ 90, which suggests that these genes are responding to growth angle but are not responsible for causing the morphologic change. The same logic can be applied to the genes of Category III, where transcript expression was different between WS and Col-0 when grown at A_gp_ 90° and when grown at A_gp_ 135° (Category III, Genes 86–128; Fig. 3c and Additional file 2: Table S3).

The genes of Category IV present a pattern of expression that indicated a potential role in skewing (Category IV; Genes 129–166; Fig. 3c and Additional file 2: Table S3), since the genes present in this category are differentially expressed between WS (which skews at A_gp_ 45°) and Col-0 (which does not skew). Some of the genes identified in this category include *HIS1-3*, *SKU5 SIMILAR 15* (*SKS15*; At4g37160; involved in oxidoreductase activity and copper ion binding), and *XYLOGLUCAN ENDOTRANSGLUCOSYLASE/HYDROLASE 26* (*XTH26*; At4g28850; involved in hydrolase activity and cell wall remodeling). *SKS15* is related to a known SKU gene (*SKU5*), which is also known to have substantial impact on root growth and morphology [56]. Protein products of *XTH26* reduce cell wall elongation in roots with altered root hair morphology [59], which may also impact the overall directionality or skewing of the root.

The second category of genes whose differential expression between WS and Col-0 indicated a role in skewing (Category V; Genes 167–192; Fig. 3c and Additional file 2: Table S3) contains genes that were differentially expressed between the ecotypes at both A_gp_ 45° and 135. The transcription of these genes was altered in the same way at both growth angles, and since skewing occurred at both growth angles (Fig. 2), it can be assumed that these genes very likely play some role in the skewing morphology (Additional file 2: Table S3). A couple of the genes identified include *MODIFIER OF SNC1 2* (*MOS2*; At1g33520; where *SNC1* is *SUPPRESSOR OF NPR1-1, CONSTITUTIVE* and *NPR1-1* is *NONEXPRESSER OF PATHOGENESIS-RELATED 1*; At4g16890 and At1g64280, respectively) and *BEL1-LIKE HOMEODOMAIN 10 (BEL10*; At1g19700, where *BEL1* is a homeodomain transcription factor controlling ovule patterning; At5g41410). *MOS2* is a DNA-binding gene that plays a role in the immune response pathway and in microRNA (miRNA) maturation [60]. miRNAs are heavily involved in gene regulation [61]. Since Col-0 roots have higher transcript levels of *MOS2* at both A_gp_ 45° and 135° than WS, it is possible that miRNAs are involved early in the signaling pathway leading toward root growth parallel with gravity. The lower levels of *MOS2* in WS roots could be limiting the rate of signal transduction, changing the entire pathway, and ultimately changing root directionality and introducing root skewing. *BEL10* was upregulated in Col-0 roots compared to WS roots grown at A_gp_ 45° or 135° (Additional file 2: Table S3). *BEL10* is a transcription factor that interacts with PLP, a blue light receptor also involved in response to salt or dehydration stresses [62]. Higher or lower intracellular levels of phosphate could alter the ability of this gene to interact downstream in phosphate signaling pathways [36]. Additionally, inorganic phosphate depravation can influence seemingly redundant signal peptides to subsequently alter root growth [63], which may, in turn, alter overall root directionality and impact skewing.

The third category of genes that indicated a role in skewing (Category VI; Genes 193–256; Fig. 3c and Additional file 2: Table S3) contains genes that were differentially expressed between Col-0 and WS when roots were grown at A_gp_ 135°. Since WS roots skewed at this growth angle and did not wave differently from roots grown at A_gp_ 90°, the genes identified in this category were considered likely to be associated with skewing. Some of the genes in this category include *RESPONSIVE TO ABA 18* (*RAB18*; At5g66400) and *PINOID BINDING PROTEIN 1* (*PBP1*; At5g54490; Additional file 2: Table S3). *RAB18* was downregulated in Col-0 roots compared to WS roots at A_gp_ 135°. This dehydrin-related protein is reduced after exogenous 1-Aminocyclopropane-1-carboxylic acid (ACC) application, meaning it responds directly to ethylene in addition to abscisic acid (ABA) [64]. *PBP1* was downregulated in Col-0 roots compared to WS roots at A_gp_ 135°. Since this PINOID-binding protein is upregulated by auxin [65], the different *PBP1* levels between Col-0 and WS could indicate different auxin levels between the two ecotypes [65, 66]. Additional genes identified in Category VI will be discussed in the following section, due to their overlap with previously identified skew gene candidates.

### Eleven genes remaining across all sets are most likely to be involved in skewing in various processes

A subset of most likely candidate genes was identified by the intersection of the list based on growth angle in WS and the list based on comparative gene expression between WS and Col-0 (Fig. 4), with the overlap of the two lists indicated in the Venn diagram, found in Fig. 4a. The list of 92 genes based on growth angle of WS (Fig. 4b; left heat map labeled “Physiology”) was compared to the list of 128 genes identified between Col-0 and WS (Fig. 4b; right heat map labeled “Genotype”), producing 16 genes that appear in both lists (Fig. 4a and b; Y* in both Additional file 1: Table S2 and Additional file 2: Table S3). The majority of these 16 genes are found involving A_gp_ 135°, shown in bold text and dark lines. A_gp_ 135° was the growth condition that elicited root skewing with minimal waving in WS, so to have the majority of overlapping genes identified by both the physiological and genotypic comparisons represented in this A_gp_ provides more support for their involvement in root skewing. These 11 genes are noted as the highly probable skew gene candidates (HPSGC).

The 11 HPSGC are involved in many different processes and were subjected to a thorough literature and expression map search using many available databases, such as Suba3, TAIR, and the Arabidopsis eFP browser [67–71]. These genes were searched with the goal of finding associations with cell expansion, division, auxin transport, or any process involved with root tropisms, so as to develop insights into their roles in skewing. Sub-organ localizations (e.g. columella cells, root tip, root hairs, etc.) were noted where possible. A summary of these results is found in Table 2.

- *MIOX4* is expressed in root hairs, stele, and lateral root cap [71], and digested columella cells have lower transcript abundance in response to auxin [70]. Additionally, overexpression of *MIOX4* in the presence of l-Ascorbic acid have increased root growth [72]. *MIOX4* influences root growth responses during stressed conditions [73], but knockouts do not have abnormal cell walls, despite the role of *MIOX4* upstream of polysaccharide integration into the cell wall [74].
- *PAP24* is not highly expressed in any root zones [69–71], but is predicted to be in plant cell walls and to have acid phosphatase and metal ion binding activity [67].
- *ASN1* is expressed in root hairs [71] and in the root under phosphate starvation [75]. *ASN1* is also responsive to darkness and sucrose starvation, and may have a role in response to viral infections [76].
- *At2g25150* is highly expressed in the cortex under normal conditions [71], but is not shown to be auxin responsive [70]. In the literature, At2g25150 is described to have a product that is a BAHD enzyme with spermidine coumaroyl CoA acyltransferase activity in roots [77] and is upregulated by cytokinin activity [78], which is evidence for its activity in cell division [77].
- *SIS* is not significantly expressed in roots under normal conditions, but is upregulated in the columella cells, root cap, and epidermis of Arabidopsis roots after 1 h of salt stress [79] and 1 hour at low pH [80]. *SIS* is also known to be involved in salt tolerance [67].
- *At5g66780* is not expressed in the root under normal conditions, but is upregulated in the root tip at low pH levels and under salt stress [74, 80]. Under normal conditions, *At5g66780* is expressed in all zones along the root epidermis, though not contiguously [71]. This gene is unknown; no other published information exists on this gene.
- *At3g28310* is expressed in the procambium of the root in the elongation zone [69, 71]. This gene produces a hypothetical protein with DUF677 [67] and is not well studied.
- *DIN2* is not expressed in the root under normal condtions, but is upregulated in roots under salt stress [79] and in conditions lacking inorganic phosphate [75]. *DIN2* mRNA can be transported from cell to cell [81], and is involved in a process that can lead to weakened root cell walls when under salt stress [82].
- *SEN1* is expressed in mature and developing root hairs [71], and is downregulated in the presence of auxin [70]. *SEN1* is regulated by both methyl-jasmonate and salicylic acid [83], and strongly induced by phosphate starvation [84],. Knockout mutants are growth deficient in the light [67].
- *SWEET11* is present in the vasculature in the zone of maturation [71], and is downregulated in the cortex of sulfur-deficient roots after 24 h [80]. *SWEET11* is a known sucrose efflux transporter that can assist with carbon transport to the roots in times of water stress [85].
- *HKT1* is expressed in the proto and metaphloem of the zone of maturation in Arabidopsis roots [69, 71], and is linked to salt and water stress responses [86]. Knockout *hkt1* has significantly lower root sugars and higher tricarboxylic acid following salt stress, indicating a role for sugar metabolism in salt stress [86]. HKT1 is also regulated by cytokinin [87], and has a close interaction with ABSCISIC ACID INSENSITIVE 4 (*ABI4*; At2g40220), where both gene products are expressed in the same cells and *ABI4* binds to sites in the HKT1 promoter [88].

**Fig. 5:**
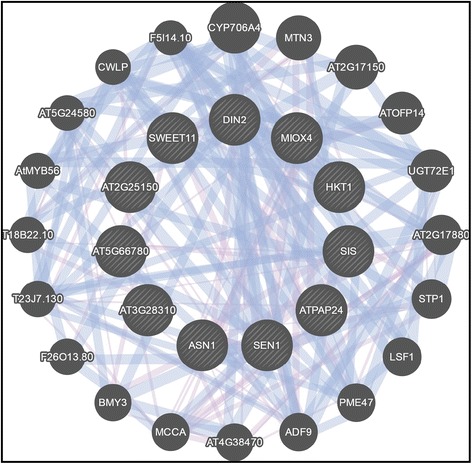
Co-expression and co-localization network of HPSGC shows each member is linked with others. Additionally, other genes pulled into the network are other members of the same signaling networks or downstream cell wall remodeling. Network generated with GeneMANIA [119]

The 11 HPSGC show similar expression patterns in that none are particularly expressed in the root tip versus the rest of the root and are all closely related into one co-expression and co-localization network (Fig. 5), despite being members of disparate signaling pathways and potential activities (Table 2). These different signaling and environmental sensing pathways, such as salt, sugar, and darkness responses, are linked by their individual gene members. For example, *ASN1*, *SWEET11*, and *HKT1* are involved in sucrose response, sugar transport, and salt signaling, respectively. These three genes, which are not co-expressed or co-localized with one another, are all co-expressed with a common gene (*SWEET12*; Fig. 5), which further complicates the roles of each gene involved. Other HPSGC are involved with more downstream processes. DIN2 and SIS are involved in two different environmental sensing and cell signaling processes, are co-localized with one another, and are both co-localized with ACTIN DEPOLYMERIZING FACTOR 9 (ADF9; At4g34970) and PLANT INVERTASE/PECTIN METHYLESTERASE 47 (PME47; At5g04970; Fig. 5), both of which are involved in cell wall remodeling. Other genes pulled in by this co-expression and co-localization network are related to handling sugar signaling downstream of sensing, such as *SUGAR TRANSPORTER 1* (*STP1*; At1g11260) and *LIKE SEX4 1* (*LSF1*; At3g01510, where *SEX4* is a plant-specific glucan phosphatase; Fig. 5). An additional network file shows the relationship between HPSGC and these additional genes [see Additional file 4]. This computational approach to the HPSGC illustrates possibilities for how these environmental sensing and signaling pathway genes could be involved in the generation of root skewing.

Sugar signaling is a major signaling pathway identified in the skew gene candidates. Sucrose efflux transporters *SWEET11* and *SWEET12* were both upregulated in WS roots grown at A_gp_ 45° and 135°. *SWEET11*, a member of the HPSGC, was also upregulated in Col-0 roots grown at A_gp_ 45° (Table 1 and Additional file 1: Table S2). These genes are also associated with response to salt stress, divalent metal ion transport, and are integral in the endomembrane system [89, 90]. SWEET11 and SWEET12 are shown to assist in phloem unloading into the apoplast [91], with SWEET12 responding to CO_2_ as well [92]. This sugar transport could be used for signaling, as other members of the *MtN3*/saliva/*SWEET*-family of genes are involved with ion transport and physiological process regulation [93]. Root exudates are also involved in regulation of the surrounding microbiome and microenvironment [94], and these SWEET efflux proteins may be involved in shaping root growth and directionality through signaling. Additionally, *ASN1* was upregulated in WS roots grown at A_gp_ 135° and was downregulated in both Col-0 and WS roots grown at A_gp_ 45° (Table 1 and Additional file 1: Table S2). ASN1 responds to sucrose starvation, and mRNA accumulation is suppressed with sugar in some tests [95, 96]. Additionally, both of these genes are responsive to absence of light, and may be involved in light/sugar signaling pathways (Additional file 1: Table S2). It is possible that ASN1 would be involved with the aforementioned *SWEET* family network. Both *DIN2* and *ASN1* were upregulated in the presence of various heavy metals, further implicating their roles in environmental signaling pathways [97], with ASN1 being expressed in the elongation and maturation zones in the root [71].

Other genes in the HPSGC are involved in sugar signaling and subsequent related phosphatase pathways. *DIN2* is a dark inducible responsive to sugar starvation [96, 98]. *DIN2* transcript is increased in the absence of type 1 and 2A protein phosphatases [96]. Though technically a metallo-phosphoesterase and a type 5 acid phosphatase, *PAP24* is a member of the HPSGC and may be involved in similar signaling pathways [99]. *MIOX4* is suppressed in the shoot by exogenous application of glucose, which my influence root expression through InsP_3_ signaling cascades [73], which itself plays a role in response to gravitropic stimuli [100]. *SEN1* is another gene induced in roots by phosphate starvation and induced in leaves by a glucose transport inhibitor [101]. *SEN1* itself may provide a link between glucose signaling and phosphate signaling [101], and is highly expressed in the meristematic zone of the root [71].

Salt signaling is another functional group identified in the HPSGC. *HKT1* has a role in salt signaling in Arabidopsis roots [102], and is likely involved in loading sodium ions into phloem to transport to roots, indirectly regulating potassium ion concentrations [86]. *HKT1* activity is reduced by high calcium levels [103, 104], which could point to a crosstalk linkage point between calcium and salt signaling pathways. *SIS* also has a role in salt tolerance, but is mostly unknown at this time [105]. Some of these salt-related genes are present in root regions that would imply signaling activity and growth determination. For example, *DIN2* is present in the columella cells and stele of roots under salt stress, while *SIS* is present in the epidermis, lateral root cap, cortex, and partially present in the endodermis [79].

## Conclusions

This work investigated the transcriptional differences between skewing and non-skewing roots. Comparisons within WS revealed genes that responded to the angle of growth (A_gp_) during the process of skewing. These genes were cross referenced with transcripts differing between the WS and Col-0 genotypes to refine the list of genes that are most probably be involved in root skewing. A majority of the highly probable skew gene candidates (HPSGC) are directly associated with environmental sensing (e.g. salt, sugar, hormones, darkness), upstream of physical growth differences (e.g. cell wall remodeling, cell division, cell elongation). Thus, pathways that respond to disparate signals from the root local environment may drive the root behavior of skewing. However, it is also possible that some of the HPSGC are responses to the altered phenotype, rather than the basis of the phenotype. In order to separate these two hypotheses, future studies could investigate the HPSGC to find the specific pathways and molecular mechanisms contributing to root skewing.

## Methods

### Plants, treatments, and morphometric assays

*Arabidopsis thaliana* plants (wild type cultivars Col-0 and WS) were grown on media plates made from 0.5x MS liquid media, autoclaved with 0.5% Phytagel and poured in square-gridded plates (Fisherbrand, Fisher Scientific, Pittsburgh, PA). Seeds were wet sterilized in 1.7 mL Eppendorf microfuge tubes (Eppendorf, Hamburg, Germany) using a 5-min 70% ethanol wash, followed by a 5 min 50% v/v sodium hypochlorate solution wash (8.3%; Clorox, Oakland, CA), followed by 6 washes with sterile ddH_2_O. Seeds were planted on plates and moved to 4 °C for 2 days, followed by three days of vertical growth (A_gp_ 90°) in 19 °C +/- 2 °C, and 24-h fluorescent light at approximately 80 μmol m^-2^ s^-1^ PAR. Plates were photographed, moved to their respective experimental condition (A_gp_ 45°, 90°, or 135°), and photographed again on day 8 after germination (day 5 after gravistimulation). Plants were harvested and fixed in RNAlater (Ambion, Grand Island, New York, USA). Images of 8 day old plates were stacked, aligned, and measured using JFilament plugin for ImageJ [106–108]. Root measurements were processed through a custom R script, available on GitHub [109]. Data were analyzed using R and two-way ANOVAs with Type II sum of squares [110]. Post hoc analysis was conducted using Scheffé’s method.

### RNA and microarray

Roots were dissected from shoots and RNA was extracted using Qiagen RNeasy Plant Mini Kit (Qiagen, Hilden, Germany). Five roots were used for each chip, and three chips were used per condition. Lateral roots were not quantified, but did not appear to be significantly different between treatments. Initial RNA concentration was determined by Eppendorf BioSpectrometer (Eppendorf, Hamburg, Germany). Final RNA concentration was determined on a NanoDrop Spectrophotometer (NanoDrop Technologies Inc., Wilmington, DE) and sample quality was assessed using the Agilent 2100 Bioanalyzer (Agilent Technologies Inc., Santa Clara, CA). Briefly, 100 ng of total RNA from each sample was reverse transcribed into double-stranded cDNA, from which biotin-labeled cRNA was generated using the 3′ IVT plus Kit (Affymetrix, Santa Clara, CA). The cRNA was purified using magnetic beads and was fragmented. Following fragmentation, cRNA products (12.5 μg) were hybridized with rotation to the Affymetrix GeneChip® Arabidopsis ATH1 Genome Arrays for 16 h at 45 °C. Arrays were washed on a Fluidics Station 450 (Affymetrix, Santa Clara, CA) using the Hybridization Wash and Stain Kit (Affymetrix, Santa Clara, CA) and the Washing Procedure FS450_0004. Fluorescent signals were measured with an Affymetrix GeneChip Scanner 3000 7G. Initial data analysis was carried out using the MAS5 algorithm within the Affymetrix Expression Console software. Microarray experiments were performed at the Interdisciplinary Center for Biotechnology Research Microarray Core, University of Florida. The datasets supporting the conclusions of this article are available in the Gene Expression Omnibus repository [GSE83242].

### Data processing, comparison tools, and qRT-PCR validation

Data were normalized using RMA algorithm using the Limma and Bioconductor packages in R. Differential analyses were processed using R and the Limma package in Bioconductor. Data were imported and organized in Excel (Microsoft Corporation, Redmond, WA). Gene transcripts were significant if absolute value of the fold change was greater than 1 in a base 2 logarithmic scale, as well as a raw *p*-value cutoff of *p* < 0.05. All genes meeting these criteria were considered, mitigating the risk of false positives with the benefit of identifying as many genes as possible. False discovery rate (FDR)-correction was performed for further statistical power, with *q* < 0.05 being indicated in Table 1, Additional file 1: Table S2 and Additional file 2: Table S3. For comparisons between Col-0 and WS cultivars, genes with altered transcripts in all three growth environments were removed if the change was near the same magnitude, within ± 1 fold change (base 2 log scale). Heatmaps were generated using Gene-E (v. 3.0.204, Broad Institute, Cambridge, MA). Gene data was researched using g:Profiler [89, 90], agriGO [111], ATTED-II [112], Biogrid [113, 114], UniProt [115], KEGG [116, 117], and STRING [118] online databases. Additional visualization of gene networks was created using GeneMANIA [119].

For qRT-PCR validation of transcriptome microarray data, 460 ng of total RNA were reverse transcribed into cDNA using High Capacity RNA to cDNA Master Mix (Applied Biosystems, Foster City, CA, USA). Primers used were *PAP24* (F: 5’ – ACACGATTGGAGAGAAGGCA – 3’; R: 5’ – AACCAAGGACACGATGAGCT – 3’), *SEN1* (F: 5’ – AGGAAATGTTGCAGCAGAGG – 3’; R: 5’ – CGTTGATGGCTCTAGTCGGA – 3’), *ASN1* (F: - GGAATATTTGGGGACGGTGC – 3’; R: 5’ – CGGGACATCAAGAACATCGG – 3’), and *HKT1* (F: 5’ – TCTTGGAGTGACGGTGCTAG – 3’; R: 5’ – CAGAGGTCCATTCAAAGGCG – 3’). The cDNA was analyzed by qRT-PCR using SYBR Green reagents and was normalized to *UBQ11* prior to the internal vertical control comparison or the Col-0 to WS comparison.

## Additional files

Additional file 1: Table S2. Comparing different growth angles to vertical within WS. (XLS 25 kb)
Additional file 2: Table S3. Comparing Col-0 to WS at different growth angles. (XLS 103 kb)
Additional file 3: Validation of microarray data using qRT-PCR. The quantitative RT-PCR data for the genes encoding SEN1, ASN1, HKT1, MIOX4, SIS, SWEET11 and DINare provided numerically in a spread sheet. (XLS 12 kb)
Additional file 4: A GeneMania network of the HPSGC genes. Co-expression and co-localization network of HPSGC showing how each HPSGC member pulled in additional signaling or cell wall remodeling genes working downstream. (PDF 463 kb)

## Abbreviations

ABA: Abscisic acid
ACC: 1-Aminocyclopropane-1-carboxylic acid
A_gp_: Angle of the growth plate
Col-0: Columbia-0
DUF: Domain of unknown function.
GSA: Gravitropic set-point angle
GTP: Guanosine triphosphate
HGI: Horizontal growth index
HPSGC: Highly probable skew gene candidates
ISS: International space station
miRNA: MicroRNA
STR: Straightness
WD: Wave density
WS: Wassilewskija
