# Supplementary material for: Skewing in Arabidopsis roots involves disparate environmental signaling pathways
Source: BMC Plant Biol. 2017 Feb 1;17:31. doi: 10.1186/s12870-017-0975-9 (PMC5286820; doi:10.1186/s12870-017-0975-9)
Supplement: Additional file 4: — A GeneMania network of the HPSGC genes. Co-expression and co-localization network of HPSGC showing how each HPSGC member pulled in additional signaling or cell wall remodeling genes working downstream. (PDF 463 kb) [file 12870_2017_975_MOESM4_ESM.pdf]

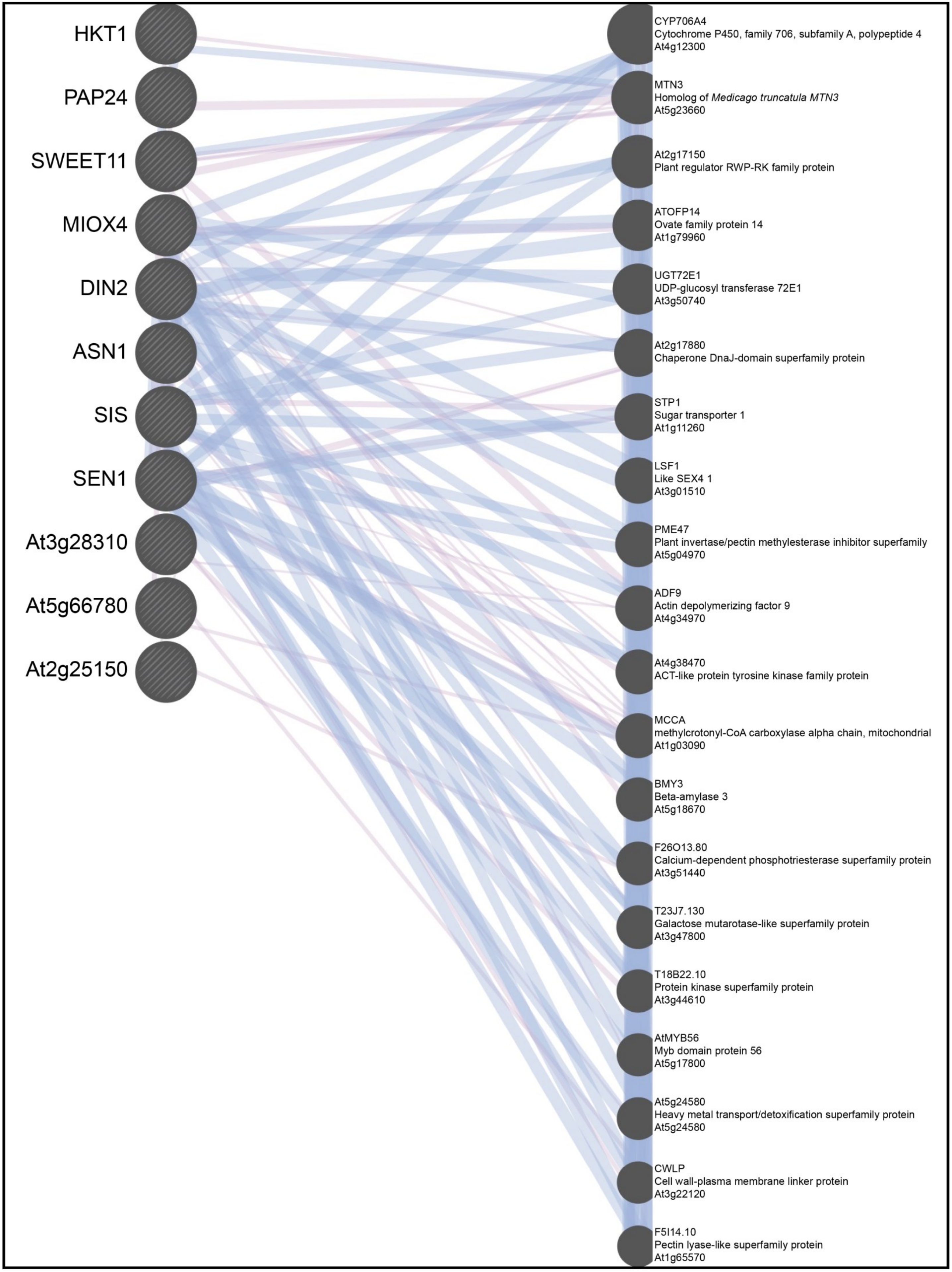

Additional file 4. Co-expression and co-localization network of HPSGC showing how each HPSGC member pulled in additional signaling or cell wall remodeling genes working downstream. Downstream genes (right column) are shown with annotation. Network generated with GeneMania.
